# Prevalence of pathogenic trypanosomes in anaemic cattle from trypanosomosis challenged areas of Itezhi-tezhi district in central Zambia

**DOI:** 10.1186/s13071-015-1260-0

**Published:** 2015-12-15

**Authors:** Njelembo J. Mbewe, Boniface Namangala, Lungowe Sitali, Ilse Vorster, Charles Michelo

**Affiliations:** Department of Public Health, School of Medicine, University of Zambia, P.O. Box 50110, Lusaka, Zambia; Department of Paraclinical Studies, School of Veterinary Medicine, University of Zambia, P.O. Box 32379, Lusaka, Zambia; Department of Veterinary Tropical Diseases, Faculty of Veterinary Science, University of Pretoria, Private Bag X04, Onderstepoort, 0110 South Africa; Tsetse and Trypanosomiasis Control Section, Department of Veterinary Services, P.O. Box 350001, Chilanga, Zambia

**Keywords:** African trypanosomosis, Cattle, Packed cell volume

## Abstract

**Background:**

The measure of anaemia status using packed cell volume (PCV) can be a reliable indicator of African trypanosomosis (AT) in the absence of other anaemia-causing conditions. However, studies that have estimated prevalence of anaemia in cattle from AT endemic areas have rarely reported the prevalence of the disease in the anaemic cattle. Therefore we investigated the prevalence of AT in anaemic cattle at sites that had recently reported the disease in Itezhi tezhi district of central Zambia.

**Methods:**

During a survey, blood samples were collected from 564 randomly selected cattle for anaemia determination from seven crush pens (Mutenda, Kapulwe, Banachoongo, Itumbi, Iyanda, New Ngoma and Shinampamba). At a PCV- value cut off of 26 %, all samples positive for anaemia were subjected to both parasitological examination on thick and thin blood smears and polymerase chain reaction-restriction fragment length polymorphism (PCR-RFLP) for detection of trypanosome DNA. Fisher’s exact test and a mixed effect logistic regression analyses were used to determine and measures associations, respectively.

**Results:**

Of 564 cattle screened, 58 (10.3 %; 95 % CI: 7.8–12.8 %) had anaemia. PCR-RFLP results showed that 17 (29.3 %; 95 % CI; 17.2–41.4 %) anaemic cattle were positive for pathogenic trypanosomes compared to 1 (1.7 %; 95 % CI: 0.0–5.2 %) on parasitological examination using thick smears. The infections were caused by *Trypanosoma congolense* and *Trypanosoma vivax*. Fisher’s exact test showed a strong association between PCV and pathogenic trypanosome infection (*P* = 0.004). A mixed effect multivariate logistic regression showed that a one unit increase in PCV reduced the likelihood of detecting AT with PCR-RFLP by 24.7 % (95 % CI: 4.6–40.6 %; *P* = 0.019) in anaemic cattle, taking into account their age and sex, with random effects for crush pen.

**Conclusion:**

These results suggest that *T. congolense* and *T. vivax* could be important causes of anaemia in cattle reared in AT endemic areas of Itezhi tezhi in Central Zambia. This also suggests that even though pathogenic trypanosomal infection was strongly associated with PCV, it could only account for up to 41 % of the anaemia in cattle. Therefore further investigation to ascertain other factors responsible for anaemia in AT endemic areas of Itezhi tezhi in Central Zambia is needed.

## Background

African trypanosomosis (AT), also known as *nagana*, is a disease of livestock caused by unicellular parasites called trypanosomes (Protozoa, Kinetoplastida). It is transmitted by tsetse flies (*Glossina spp*.) and other biting flies. *Nagana* is characterized by a slow progressive loss of condition and appetite, fever and anaemia leading to extreme emaciation, collapse and death [1, 2] if not treated. It is estimated that 50 to 70 million animals are at risk of AT [3], representing a severe constraint to the development of the affected areas and causing losses in the agricultural sector estimated at about US$1.3 billion annually in sub-Saharan Africa [4–6].

In Zambia, the most common pathogenic trypanosome species affecting cattle are *Trypanosoma congolense* and *Trypanosoma vivax*, and to a lesser extent, *Trypanosoma brucei brucei* [7]. Even though *Trypanosoma theileri* is also commonly encountered during diagnosis, it is not known to cause any disease in cattle [8]. The tsetse fly is the main determinant of trypanosomosis and is mostly found in and around protected areas such as National Parks and Game Management Areas (GMAs) [9]. Even though human settlement is not allowed in the National park, it is permitted in the GMA. Consequently, man introduces his domestic livestock in the same physical environment that harbours tsetse flies and wildlife when he settles in GMAs. This situation has greatly contributed to the distribution and risk of AT in Southern Africa [10, 11].

Accurate diagnosis of AT is important in determining the epidemiology of the disease [12]. It also facilitates development of effective disease preventive and control strategies. A number of diagnostic tests are available for the detection of trypanosomes. Parasitological tests depend on visualization of trypanosomes in wet blood films, blood smears or buffy coat preparations with the aid of a microscope. However these methods have sensitivity as low as 37 % and so a large proportion of infections are usually missed especially where there is low parasitemia [8]. With a sensitivity of 96 %, a molecular-based technique of polymerase chain reaction- restriction fragment length polymorphism (PCR-RFLP) is one of the most sensitive and specific diagnostic method of AT [8]. Its main disadvantage is that it is more costly than direct parasitological methods because it requires sophisticated equipment and materials as well as highly skilled personnel [12, 13]. This limits its use in field veterinary laboratories in AT endemic areas. Nevertheless PCR-RFLP permits identification of parasites at levels far below the detection of the parasitological techniques [12]. On the other hand, indirect diagnostic methods such as the measure of anaemia status using packed cell volume (PCV) can be a reliable indicator of AT in the field in the absence of other anaemia-causing infections [8, 14, 15]. Determination of PCV values as a diagnostic tool of AT in cattle has been shown to have higher sensitivity and specificity compared to parasitological diagnosis [8]. For this purpose, different PCV-value cut offs of 24 and 26 % as indications for anaemia in cattle have been used to estimate AT [8]. Further, sensitivity and specificity of PCV value as a diagnosis tool for AT, at those cut offs have been reported [8]. The PCV value of 26 % has a higher sensitivity of 76 % compared to 53 % at PCV cut off of 24 % [8]. Though studies have estimated the prevalence of anaemia in cattle from AT endemic areas [7, 16, 17], prevalence of the disease in anaemic cattle has rarely been reported.

In this study, we determined the prevalence of AT in anaemic cattle using a PCV-value cut off of 26 %. We further measured the association between PCV and pathogenic trypanosome infection in anaemic cattle from AT endemic areas of Itezhi-tezhi district, central Zambia.

## Methods

### Study area and population

The survey was conducted in October 2013 in the tsetse-infested Itezhi tezhi district within Central province of Zambia [9]. With a total surface area of 13,000 km^2^, approximately 50 % of Itezhi tezhi is the Kafue National Park while the rest is either GMA or Gazette forest [9]. AT, transmitted by *Glossina morsitans centralis*, is endemic in the district, with eight out of the ten veterinary camps having previously reported the disease [9]. Drug treatments with trypanocides are the main method of AT control in Itezhi tezhi. In the study area, the major cattle breeds are the Angoni and crosses of the local breeds (Angoni, Ila and Tonga breeds) with exotic breeds. According to the 2013 first round foot and mouth disease report [18], the district has approximately over 85,000 herds of cattle with approximately 2,140 bulls, 49,386 cows (including heifers), 18,525 oxen and over 15,520 calves. The cattle are managed under the extensive system with free range grazing in the Kafue flats during the dry months (April to October) and upland in the forest during the wet months (November to March).

### Sample selection

Crush pens that had consistently reported cases of AT in the three immediate past years (2010–2013) were purposively selected as study sites [9]. Using this sampling criterion and the records from the Itezhi tezhi district veterinary office, seven crush pens (Mutenda, Itumbi, Kapulwe, Banachoongo, Iyanda, New Ngoma and Shinampamba) were selected. Sample size at each crush pen was calculated to provide 95 % confidence of detecting at least one positive case of anaemia at a prevalence of 10 % [19]. Simple random sampling using computer generated random numbers from Microsoft Excel 2007 (Microsoft Cooperation) was used to select cattle at each of the selected crush pens. All animals were given identity numbers and their characteristics on age and sex were captured on record sheets.

### Microscopy, PCV determination and buffy coat preparation

Blood samples were obtained from the ear vein of each animal into two heparinized capillary tubes. Giemsa-stained thick and thin blood smears from each animal selected were made using the first capillary tubes for microscopic examination. Using the second capillary tube, PCV values were determined as previously described [7] and all samples with equal to or less than 26 % PCV values were placed onto a labelled FTA® card (Whatman no 4, Whatman®, UK) as buffy coat spots for DNA extraction according to the manufacturer’s suggested protocol. All FTA® cards were dried and stored in envelopes at room temperature away from sunlight until [20].

### DNA extraction and PCR- RFLP

DNA was extracted from the buffy coat spots on FTA® cards using the QIAamp® DNA blood Mini Kit (Qiagen, Germany) according to the manufacturer’s instructions and eluted in 150 μl of elution buffer. The PCR-RFLP assay was performed as previously described by Geysen et al. (2003) [12]. Briefly, the PCR mixture was prepared using DreamTaq PCR Master Mix (2X) (Thermo Scientific, InqabaBiotec, South Africa). Reactions were performed in a 25 μl volume containing 12.5 μl of the DreamTaq PCR Master Mix (2x) (containing DreamTaq DNA polymerase, 2X DreamTaq buffer, 0.4 mM of each dNTP and 4 mM of MgCl_2_)_,_ 20 μM of each primer, nuclease-free water and 5 μl of DNA template. The first amplification was done using the forward primer 18ST nF2 (5’-CAACGATGACACCCATGAATTGGGGA-3’) and reverse primer 18ST nR3 (5’-TGCGCGACCAATAATTGCAATAC-3’). For the semi-nested amplification forward primer, 18ST nF2 was used with the reverse primer 18ST nR2 (5’-GTGTCTTGTTCTCACTGACATTGTAGTG-3’). The PCR conditions for the first amplification were as follows: denaturation at 95 °C for 4 min and then 40 cycles of 95 °C for 30 s, 58 °C for 45 s and 72 °C for 1 min. For the semi-nested PCR 2.5 μl of amplification product from the first run was added to the PCR mix and the amplification programme was identical to the first run except the number of cycles was reduced to 25. A negative- (PCR mixture without DNA template) and positive- (known *T. congolense*, *T. vivax*, *T. b. brucei* or *T. theileri* DNA samples) controls were included in each PCR amplification. The amplification products were examined for the presence of trypanosome DNA on a 2 % agarose gel. A 100 bp ready to use DNA ladder (Thermo Scientific, InqabaBiotec, South Africa) was included in every gel for fragment size determination. The samples were run for 45 min at 120 V in 1x Tris/Acetic acid/EDTA (TAE) buffer, stained with ethidium bromide and photographed under UV illumination using the ChemiDoc™ XRS system (BioRad, South Africa). Detection of trypanosome DNA was regarded as a positive infection. Positive products from the semi-nested PCR were digested in 15 μl reactions using the restriction enzymes *Msp*1 (Fermentas, InqabaBiotec, South Africa) and *Eco*571 (Fermentas, InqabaBiotec, South Africa) in buffer G/Tango according to the manufacturer’s instructions. Four microlitres of the restricted sample was then mixed with 2 μl of loading buffer and loaded onto a 12 % polyacrylamide gel. A 100 bp DNA ladder (Thermo Scientific, InqabaBiotec) for fragment size determination was also included. The DNA fragments were thereafter separated by gel-electrophoresis in 1x Tris/Boric acid/EDTA (TBE) buffer at 80 V for 2.5 h. The gel was then stained with SYBR Green I gel stain (Roche, South Africa) for 30 min and photographed using the ChemiDoc™ XRS system (BioRad, South Africa).

### Statistical analysis

STATA IC version 11 was used for management and analysis of data collected and prevalence determination of AT in anaemic cattle. Fisher’s exact test was used to determine association between pathogenic trypanosome infection status (presence or absence) and PCV in anaemic cattle. A mixed effects multiple logistic regression was used to measure associations with the outcome variable being AT status (from PCR-RFLP results) and the explanatory variables being PCV, animal age and sex with crush pen for random effects. P values <0.05 were considered statistically significant.

### Ethical statement

Approval to conduct this study was granted by the Excellence in Research Ethics and Science IRB number 00005948 (reference number 2013-June-003). In addition, approval and clearance for the study was obtained from the Department of veterinary authorities. Permission was sought from cattle owners to collect blood from their animals. Blood samples were collected from animals by qualified veterinary officials.

## Results

### Characteristics of cattle

From the 1, 227 cattle that were eligible and presented for blood sample collection, 564 cattle were selected, of which 161 (28.5 %; 95 % CI: 24.8–32.3 %) were males (bulls and oxen) and 403 (71.4 %; 95 % CI: 67.7–75.2 %) were females (heifers and cows). The overall mean age in years of the cattle sampled was 5.6 (95 % CI: 5.3–5.8). By sex, mean age of male cattle in years was 4.6 (95 % CI: 4.2–5.0) while for females it was 5.9 (95 % CI: 5.6–623). A comparison of the mean ages by sex showed a significant difference (*P* < 0.000), with female cattle having a higher mean age than males.

### Anaemia and trypanosome infection

Using the PCV value cut off of 26 %, 58 (10.3 %; 95 % CI: 7.8–12.8) out of 564 animals screened had anaemia (Table 1). In anaemic cattle, the PCV values ranged from 14 % to 26 % (Table 2). Microscopic examination of thick smears detected one trypanosome infection (1.7 %; 95 % CI: 0.0–5.2 %) in 58 anaemic cattle. No parasites were detected on thin smears.

**Table 1:** Prevalence of pathogenic trypanosomes in anaemic cattle at each crush pen

Crush pen	Sample size	No. of anaemic cattle (%; 95 % CI)	No. of PT positive anaemic cattle (%; 95 % CI)	No. (Trypanosome species prevalence; 95 % CI)		
				*T.c*	*T.v*	*T.c* + *T.v*
Banachoongo	115	14 (12.0; 6.1–18.2)	3 (21.4; 0.0–46.0)	1 (7.1 %; 0.0–22.6 %)	1 (7.1 %; 0.0–22.6 %)	1 (7.1 %; 0.0–22.6 %)
Itumbi	25	1 (4.0; 0.0–12.2)	0	0	0	0
Iyanda	66	1 (1.5; 0.0–4.5)	0	0	0	0
Kapulwe	89	8 (9.0; 2.9–15.0)	0	0	0	0
Mutenda	152	22 (14.5; 8.8–20.1)	13 (59.1; 36.8–81.4)	12 (54.5 %; 31.9–77.1 %)	1 (4.5 %; 0.0–14.0 %)	0
New Ngoma	42	9 (21.4; 8.4–34.4)	0	0	0	0
Shinampamba	75	3 (4.0; 0.0–8.5)	1 (33.3; 0.0–100)	1 (33.3 %; 0.0–100 %)	0	0
Total	564	58 (10.3; 7.8–12.8)	17 (29.3; 17.2–41.4)	14 (24.1 %; 12.8–34.5 %)	2 (3.4 %; 0.0–8.3 %)	1 (1.7 %; 0.0–5.2 %)

PT- Pathogenic trypanosome, *T.c*- *Trypanosoma congolense*, *T.v*- *trypanosoma vivax*, *T.c* + *T.v*- Mixed infection

**Table 2:** Association between PCV values and trypanosome infection status on RFLP-PCR in Anaemic cattle

PCV value (%)	No. of anaemic cattle tested using RFLP-PCR (%)	
	Negative PT trypanosome DNA	Positive PT trypanosome DNA
14	1 (2.4)	0 (0.0)
15	0 (0.0)	1 (5.9)
16	0 (0.0)	2 (11.8)
17	0 (0.0)	1 (5.9)
18	1 (2.4)	0 (0.0)
19	0 (0.0)	3 (17.6)
20	1 (2.4)	0 (0.0)
21	1 (2.4)	0 (0.0)
22	1 (2.4)	1 (5.9)
23	2 (4.9)	1 (5.9)
24	6 (14.6)	1 (5.9)
25	6 (14.6)	4 (23.5)
26	22 (53.7)	3 (17.6)
Total	41 (100.0)	17 (100.0)
Mean PCV value (95 % CI)	24.6 (23.8–25.4)	21.6 (19.6–23.7)
*P*-value at 95 % CI	0.004	

PT- Pathogenic trypanosome

Using PCR-RFLP, trypanosome DNA was detected from 17 (29.3 %; 95 % CI: 17.2–41.3 %) anaemic cattle. The parasitologically positive sample was also positive by PCR-RFLP with *T. vivax* as a single infection. Trypanosome infections were recorded from Shinampamba, Mutenda and Banachoongo crush pens (Table 1). The infections were caused by *T. congolense* savannah (14/58[24.1 %; 95 % CI: 12.8–34.5 %]), *T. vivax* (2/58[3.4 %; 95 % CI: 0.0–8.3 %]) and one mixed infection of *T. congolense* and *T. vivax* (1.7 %; 95 % CI: 0.0–5.2 %). According to PCR-RFLP, *T. theileri* was also detected in 4 anaemic cattle (6.9 %; 95 % CI: 0.2–13.6 %) from Banachoongo (two) and Kapulwe (two) crush pens. A strong associated (*P* = 0.004) between pathogenic trypanosome infection and PCV was observed in anaemic cattle (Table 2).

The mixed effects multiple logistic regression showed that, a one unit increase in PCV value significantly reduced (*P* = 0.019) the likelihood of detecting a pathogenic trypanosome infection with PCR-RFLP by 24.7 % and the reduction could be as low as 4.6 % and as high as 40.6 % in anaemic cattle while taking into account their age and sex with random effects for crush pen at 95 % confidence interval. Age and sex were not significantly (*P* > 0.05) associated with pathogenic trypanosome infections in anaemic cattle.

## Discussion

The present study demonstrated that a larger proportion of anaemic cattle from AT challenged areas of Itezhi tezhi were not infected with pathogenic trypanosomes. This observation is an indication that there could be other anaemia causing factors in AT challenged areas of Itezhi tezhi. Despite this, a significant association was observed between pathogenic trypanosome infection and PCV in anaemic cattle. This suggests that PCV could still be used as an indicator of AT even in the presence of other anaemia causing factors in endemic areas contrary to findings from other studies [8]. However, investigations that take into account PCV and RFLP-PCR status of both anaemic and non-anaemic cattle should be undertaken in order to verify the assertion. The large proportion of anaemic cattle not being infected with AT also implies that there is need to carry out investigations that will establish other anaemia causing factors in order to plan for effective interventions to improve animal health in AT endemic areas of Itezhi tezhi. The significant association between AT and PCV levels in anaemic cattle obtained in this study are consistent with findings from other studies that have reported anaemia prevalence in AT challenged areas [7, 8, 16, 17].

On account of the study design employed where samples from cattle were grouped at crush pens, our results could have been biased by introduction of pseudo-replication. However this grouping was addressed during the statistical analysis by using a mixed effect multiple logistic regression, with crush pen added as a random effect. The mixed effect multiple logistic regression also showed a strong association between PCV and AT status in anaemic cattle, with a reduced likelihood of AT for every unit increase in PCV.

The prevalence obtained from parasitological diagnosis was much lower than that observed from the molecular based diagnosis of PCR-RFLP. This is consistent with findings from other studies [7] and could arise on account of the low sensitivity of parasitological diagnostic methods compared to PCR-RFLP [8]. Low parasitemia could also possibly explain the low prevalence observed from the parasitological diagnosis especially that the study focused on anaemic cattle, which could have been in the chronic phase of AT. PCR-RFLP is also affected by parasitaemia. However due to its high sensitivity, the AT prevalence reported in this study is probably a good estimation in anaemic cattle of AT endemic areas of Itezhi tezhi district.

The study findings indicate that a combined use of PCV and PCR-RFLP can be valuable in investigations of chronic cases as well as establishment of presence or absence of AT in an area. This indication can only be ascertained only when the overall prevalence of AT in all the cattle sampled is established. Additionally, the results show that the majority of AT infections were caused by *T. congolense*, which is consistent with field observations from elsewhere [7, 16, 21, 22]. This observation could be due to the development process of *T. congolense* which is confined to intravascular blood [23], thereby increasing the chances of its diagnosis when blood samples are used. In contrast, *T. brucei* is less pathogenic and may traverse the walls of the blood vessels into connective tissue of the intermediate host [23]. This could explain the non detection of *T. brucei* DNA in our samples, more so that cattle with anaemia were more likely to have the chronic rather than the acute form of the disease.

## Conclusion

The study showed that despite a larger proportion of anaemic cattle not being positive for pathogenic trypanosomes upon using PCR-RFLP, the PCV was still strongly associated with AT in anaemic cattle. This shows that other anaemia causing factors could be present in AT endemic areas of Itezhi tezhi. Therefore an investigation to establish the other anaemia causing factors is recommended to control anaemia in cattle from AT endemic areas of Itezhi tezhi. Finally, the study suggests that *T. congolense*, and to a lesser extent *T. vivax*, could be the most important cause of anaemia in AT endemic areas of Itezhi tezhi.

## Abbreviations

AT: African trypanosomosis
CI: Confidence interval
DNA: Deoxy ribonucliec acid
GMA: Game management area
PCR-RFLP: Polymerase chain reaction- restriction fragment length polymorphism
PCV: Packed cell volume
PT: Pathogenic trypanosomes
